# Design of a Multistable Cantilever Piezoelectric Vibration Energy Harvester with Nonlinear Force Customization

**DOI:** 10.3390/s26154812

**Published:** 2026-07-29

**Authors:** Erfang Luo, Fazhi Li, Xiaolei Jin, Xiaoqing Zhang, Zhushi Rao, Donglin Zou

**Affiliations:** 1 School of Artificial Intelligence, Shanghai Minhang Polytechnic, Shanghai 201111, China; 2 State Key Laboratory of Mechanical System and Vibration, Shanghai Jiao Tong University, Shanghai 200240, China

**Keywords:** multistable energy harvester, bistable energy harvester, tristable energy harvester, pentastable energy harvester

## Abstract

**Highlights:**

**What are the main findings?**

- A piezoelectric cantilever energy harvester with user-specified number and coordinates of equilibrium points is proposed.
- Both tristable and pentastable harvesters are designed using a single nonlinear force customization device.

**What are the implication of the main findings?**

- The pentastable harvester achieves wider bandwidth, lower harvesting frequency, and easier inter-well motion than the tristable one.
- Structural complexity does not increase with the number of stable equilibrium points, unlike conventional spring- or magnet-based designs.

**Abstract:**

Multistable energy harvesters have attracted considerable attention due to their shallow potential wells, which facilitate low-energy inter-well oscillations. Although bistable or tristable configurations can be realized using combined magnets or springs, two critical challenges remain: (i) the difficulty in obtaining a higher number of stable equilibrium points and (ii) the inability to arbitrarily prescribe the coordinates of these equilibrium points. To address these issues, this paper proposes a piezoelectric cantilever beam-based multistable energy harvester that allows programmable specification of both the number and the positions of equilibrium points. As demonstrations, a tristable and a pentastable energy harvester with user-defined equilibrium coordinates are designed, and their energy harvesting performances are systematically investigated. Simulation and experimental results show that under an excitation acceleration of 0.1 g, both harvesters can only perform intra-well motion, exhibiting softening nonlinearity. When the excitation acceleration increases to 0.2 g, the pentastable harvester successfully overcomes the maximum potential barrier to achieve inter-well oscillation, displaying hardening nonlinearity and significantly broadening the operational bandwidth, while the tristable harvester remains confined to intra-well motion. At an excitation acceleration of 0.4 g, both harvesters can achieve inter-well oscillation, but the pentastable harvester possesses a wider operational bandwidth and a lower starting frequency for energy harvesting. The proposed method enables the design of multistable vibration energy harvesters without increasing structural complexity with the number of equilibrium points, which is of great significance for optimizing multistable vibration energy harvesters.

## 1. Introduction

In recent years, microsensors have been widely used in many fields such as environmental monitoring, the automotive industry, the Internet of Things, and wearable devices. At present, most microsensors rely on chemical batteries for power supply, which leads to issues such as environmental pollution and limited service life. To address these problems, researchers have proposed harvesting energy from the environment, including wind energy, human kinetic energy, and vibration energy, to achieve self-powered microsensors. Among these, vibration energy has attracted significant attention due to its wide availability, cleanliness, and stability [1,2,3,4,5,6,7].

However, conventional linear vibration energy harvesters suffer from a narrow response bandwidth, making it difficult to efficiently harvest the widely available broadband vibration energy in the environment. Therefore, nonlinear vibration-based energy harvesters have been extensively studied due to their wider operating bandwidth [8,9,10,11,12]. Depending on the number of stable equilibrium points, nonlinear energy harvesters can be classified into monostable [13,14,15,16] and multistable types [17,18,19]. Studies have shown that multistable energy harvesters can transition between different equilibrium states, generating large-amplitude oscillations (inter-well vibrations) [20,21,22], thus outperforming monostable harvesters. Consequently, multistable energy harvesters, including bistable [23,24,25,26], tristable [27,28,29,30], and quadstable [31,32,33,34] designs, have been continuously developed. For example, Cui et al. [35] proposed a jellyfish-inspired bistable piezoelectric–triboelectric hybrid generator that achieves low-frequency broadband energy harvesting through a low-potential barrier structure and significantly improves output power. Chen et al. [36] presented a bistable energy harvester with a self-lowering potential barrier effect, realizing broadband response via magnetic spring coupling. This structure facilitates inter-well motion, thereby yielding higher output power and wider bandwidth at low frequencies. Ma et al. [37] designed a magnetostrictive bistable energy harvester inspired by the Venus flytrap, achieving potential energy storage and release through a subsidiary beam constraint. Wang et al. [38] proposed an electromagnetic bistable energy harvester with an asymmetric potential well. By means of magnetic–elastic coupling, low-frequency large-amplitude vibration is achieved, which significantly broadens the bandwidth and enhances output power. Zhang et al. [39] designed a bistable bridge vibration energy harvester based on magnetic interaction that can provide stable output under vehicle excitation. Appropriate magnetic spacing optimizes the potential energy distribution and improves harvesting efficiency. Bai et al. [40] proposed a bistable cantilever beam-based piezoelectric–triboelectric hybrid nanogenerator that effectively enhances contact force and piezoelectric deformation. Huang et al. [41] developed a bioinspired bistable magnetic-elastic vibration energy absorber, integrating vibration suppression and energy harvesting. This structure maintains good energy conversion capability even under weak excitation. Luo et al. [42] designed a bistable triboelectric nanogenerator inspired by double-wing flight, achieving low-frequency broadband energy harvesting. Tan et al. [43] proposed an arched bistable triboelectric generator that harvests low-frequency energy through nonlinear stiffness. Xiong et al. [44] introduced an “M”-shaped bistable piezoelectric beam structure that improves power generation performance while reducing stress concentration, resulting in good fatigue resistance and output capability. Dang et al. [45] designed a T-shaped bistable piezoelectric energy harvester with a moving magnet, achieving multi-peak resonance and broadband response. Li et al. [46] proposed a bistable piezoelectric device integrating vibration absorption and energy harvesting, showing excellent performance in both vibration suppression and energy harvesting. Man et al. [47] developed a bistable piezoelectric harvester with a dynamic amplifier, significantly improving the output performance under low excitation through parameter optimization. Zhang et al. [48] presented a 3D-printed asymmetric rail bistable piezoelectric harvester for ultra-low frequency vibration energy harvesting. Qaseem et al. [49] introduced magnetic nonlinearity to achieve bistable characteristics of a triboelectric energy harvester, effectively broadening the working bandwidth. Rezaei et al. [50] conducted a comparative study of monostable, bistable, and tristable nonlinear energy sinks, noting that multistable structures have advantages in broadband energy harvesting, while bistable designs are more efficient in energy conversion. Gatti et al. [51] proposed a tunable nonlinear stiffness device capable of realizing multiple response forms from monostability to tristability, offering a flexible design approach for multistable energy harvesting systems. Ma et al. [52] investigated the nonlinear dynamic characteristics of an asymmetric tristable energy harvester and found that it more readily undergoes inter-well motion. Tan et al. [53] proposed a magnetic negative-stiffness tristable triboelectric nanogenerator, achieving low-frequency large-amplitude vibration response. Zeng et al. [54] proposed a tristable nonlinear energy sink for vibration absorption and analyzed its energy dissipation mechanism. Wang et al. [55] presented an asymmetric tristable magnetic-elastic system that realizes potential energy regulation through spring compression and rotation. This structure reduces the inter-well threshold and improves energy harvesting efficiency. Fu et al. [56] proposed a quadstable energy harvester based on a nonlinear pendulum for ultra-low frequency random vibration energy harvesting. Wang et al. [57] designed a bioinspired quadstable piezoelectric energy harvester that achieves broadband response through a “snap-through” transition mechanism, showing high energy output capability under low-frequency conditions. Wang et al. [58] proposed a tunable multistable bioinspired structure that can switch among different stable states via gear adjustment, exhibiting excellent performance under low-frequency and low-amplitude excitation. Yan et al. [59] exploited geometric nonlinearity of springs to construct a quadstable piezoelectric energy harvester with multiple motion modes. Nadertehrani et al. [60] studied a quadstable piezoelectric cantilever beam system that significantly broadens the frequency band through inter-well motion.

Nevertheless, the design of multistable energy harvesters still faces two major challenges: (1) Difficulty in designing harvesters with a high number of stable states. As the number of stable states further increases, the construction complexity of the system’s potential energy function increases significantly. More nonlinear elements or coupling mechanisms are typically required to realize multi-well characteristics, which considerably raises the difficulty of design and implementation. (2) Inability to independently design the coordinates of equilibrium points. In multistable harvesters, altering the structural parameters causes all equilibrium point coordinates to change simultaneously, and the extent of change varies for each point, making coordinated regulation difficult.

To address these issues, this paper develops an improved piezoelectric energy harvester based on our proposed nonlinear force customization technique [61]. A piezoelectric cantilever beam-based multistable vibration energy harvester with user-specified equilibrium points is proposed. As examples, a tristable energy harvester (TEH) and a pentastable energy harvester (PEH) are designed. Simulations and experiments confirm the feasibility and effectiveness of the proposed approach. The experimental and simulation results reveal several key features. Under weak excitation (e.g., 0.1 g), the multistable system is dominated by softening nonlinearity and can only undergo intra-well motion. As the excitation increases (e.g., 0.4 g), the system overcomes the potential barrier to perform inter-well jumping and exhibits hardening nonlinearity. When the acceleration is sufficiently large for both harvesters to undergo inter-well vibration, the PEH displays a wider bandwidth but lower peak power, whereas the TEH shows a narrower bandwidth but higher peak power. Furthermore, it is found that appropriately increasing the number of equilibrium points lowers the critical excitation amplitude required for inter-well jumping, which provides a general design guideline for broadband energy harvesting in low-excitation environments. The remainder of this paper is organized as follows. Section 2 describes the design principle and the improved mechanism. Section 3 presents a TEH and PEH as examples. Section 4 reports simulations and experiments, showing that the PEH can reduce the potential barrier, trigger inter-well motion at a lower excitation level, broaden the operating bandwidth, and improve low-frequency energy harvesting for sensor power supply. In addition, the PEH shows potential for low-frequency energy harvesting for low-duty cycle sensor power supply. The main conclusions are summarized in Section 5.

Compared with our previous programmable equilibrium point harvester [62], the major structural difference is the arrangement of the raceway–roller mechanism. In the previous design, the raceway was integrated with the linear guide mechanism, and the roller was attached to the cantilever beam. Because the raceway and guide components had a relatively large moving mass, the auxiliary mass spring subsystem formed with the pre-compressed spring could have a low natural frequency and could couple with the cantilever vibration, thereby reducing the accuracy of the designed nonlinear force. In the present design, the roller is placed on the linear guide mechanism, and the raceway is fixed to the cantilever beam. The moving mass associated with the pre-compressed spring is therefore reduced. In addition, the auxiliary natural frequency is increased, and the coupling with the cantilever vibration is weakened. This improved configuration is expected to provide more accurate and stable nonlinear force customization.

## 2. Design Principle of Multistable Energy Harvesters

**Figure 1:**
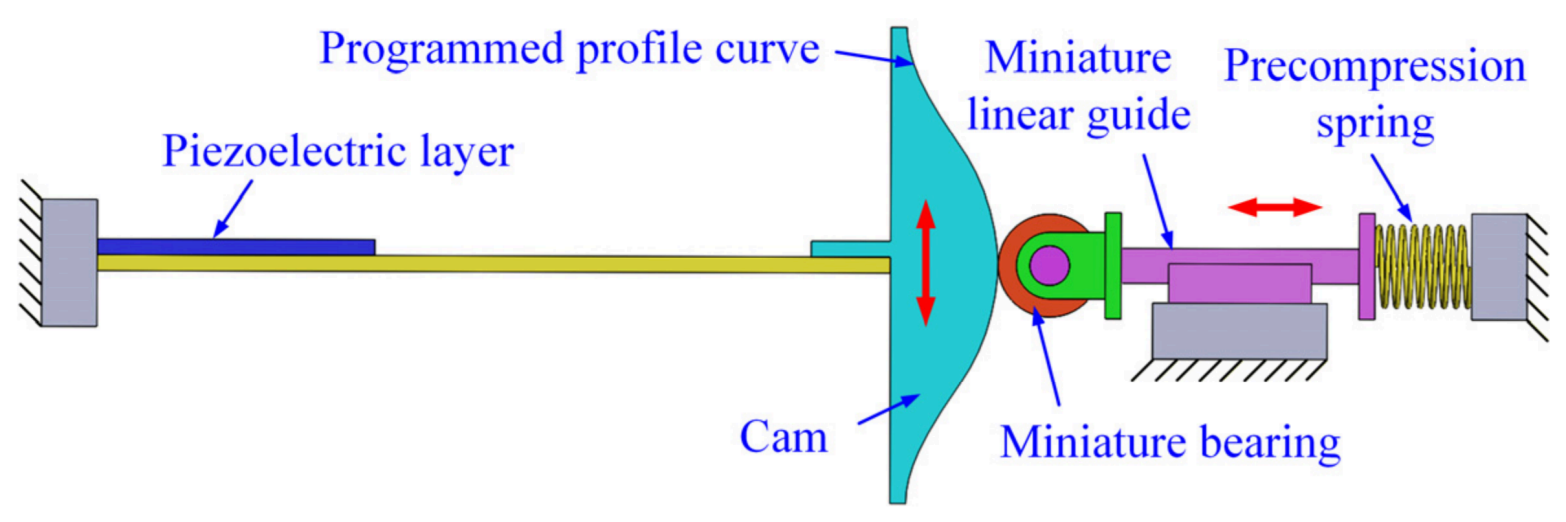
Schematic diagram of an energy harvester with multistable equilibrium points.

Figure 1 illustrates the proposed energy harvester enabling user-defined equilibrium points. The system comprises a piezoelectric cantilever beam, a contoured raceway fixed to the beam, and a nonlinear force customization module composed of a miniature roller, a linear guide, and a pre-compressed spring. The roller is constrained by the linear guide and remains in contact with the raceway. When the cantilever vibrates, the raceway profile converts the beam displacement into a prescribed spring deformation, thereby generating the required nonlinear restoring force. By tailoring the raceway contour, both the total number and the exact coordinates of stable equilibrium points can be specified according to practical requirements.

This arrangement is different from the previous design in which the raceway was mounted together with the linear guide mechanism and the roller was attached to the cantilever beam. In that configuration, the relatively large raceway-guided mass and the pre-compressed spring formed a low-frequency auxiliary mass spring subsystem that could couple with the cantilever vibration and make the actual nonlinear force deviate from the designed force. In the present configuration, the moving mass of the auxiliary subsystem is much smaller because only the roller and guide-related moving parts participate in the spring motion. Consequently, the auxiliary natural frequency is increased, the undesired dynamic coupling is reduced, and the nonlinear force can be controlled more accurately.

Given that the mass of the miniature rolling bearing is negligible, its inertial effect and the rolling friction between the bearing and the raceway are both ignored. The governing electromechanical equation of the proposed piezoelectric cantilever energy harvester can be expressed as follows [62]:

(1) $$\begin{array}{c} M\ddot{q}+F(q)+C\dot{q}-\theta V=F_{b}(t)\\ \theta \dot{q}+C_{p}\dot{V}+\frac{V}{R_{l}}=0 \end{array}$$

where q denotes the tip displacement of the cantilever beam; M is the equivalent mass of the cam and the cantilever beam; C is the equivalent damping; θ is the equivalent electromechanical coupling coefficient; $C_{p}$ is the effective capacitance; V is the output voltage across the load; $R_{l}$ is the load resistance; and $F_{b}(t)=mA_{b}\omega^{2}\cos(\omega t)$ is the base excitation force, with m being the equivalent excitation mass, $A_{b}$ the excitation amplitude, and ω the excitation frequency. F(q) is the resultant nonlinear force in the vibration direction, which can be expressed as follows:

(2) $$F(q)=K_{1}q+K_{2}q^{3}+F_{h}(q)$$

where $K_{1}$ is linear stiffness of the cantilever beam; $K_{2}$ is the geometric nonlinear stiffness of the cantilever beam; and $F_{h}(q)$ is the component force in the vibration direction resulting from the bearing–cam interaction, which can be obtained using the potential energy principle.

(3) $$U(q)=\frac{K_{h}{\left(x_{0}+S(q)\right)}^{2}}{2}-\frac{K_{h}x_{0}^{2}}{2},\;F_{h}(q)=\frac{dU(q)}{dq}=K_{h}\left(x_{0}+S(q)\right)S^{\prime }(q)$$

**Figure 2:**
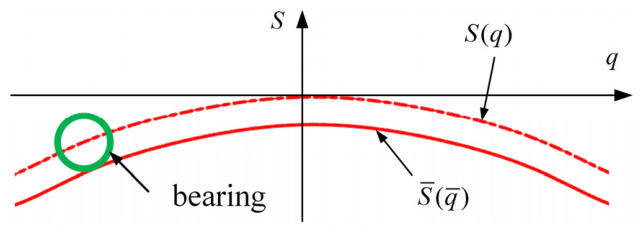
Schematic diagram of relationship between S(q) and $\bar{S}(\bar{q})$.

Here, U(q) is the potential energy of the pre-compressed spring (assuming the initial potential energy is zero). $K_{h}$ and $x_{0}$ are the stiffness and the pre-compression displacement of the pre-compressed spring, respectively. Let $\bar{S}(\bar{q})$ denote the actual profile curve of the raceway to be machined, and let S(q) denote the trajectory of the bearing center as the rolling bearing moves along the raceway. Since the bearing has a finite radius $r_{b}$, the bearing center does not trace the raceway profile directly; instead, it follows a curve that is offset from the raceway profile by a distance equal to $r_{b}$ along the normal direction at each point. This geometric relationship is schematically illustrated in Figure 2. Mathematically, the relationship between the raceway contour $\bar{S}(\bar{q})$ and the bearing-center trajectory S(q) is governed by the following offset equations:

(4) $$\bar{q}=q+r_{b}\frac{\partial S(q)}{\partial q}\sqrt{\frac{1}{1+{\left(\partial S(q)/\partial q\right)}^{2}}},\;\bar{S}(\bar{q})=S(q)-r_{b}\sqrt{\frac{1}{1+{\left(\partial S(q)/\partial q\right)}^{2}}}$$

where q and $\bar{q}$ are the horizontal coordinates of the bearing center and the raceway contact point, respectively. The first equation relates the horizontal positions, while the second equation relates the vertical positions. The term ∂S(q)/∂q represents the local slope of the bearing center trajectory, from which the unit normal vector is determined.

For an *n*-stable system, the nonlinear force F(q) can be expressed in a factored form based on its equilibrium point coordinates. Specifically, if the system has $\left(n-1\right)$ pairs of nonzero equilibrium points at $\pm a_{1}$, $\pm a_{2}$, ⋯, $\pm a_{n-1}$, together with a central equilibrium point at q=0, then F(q) can be written as follows:

(5) $$F(q)=k\prod_{i=1}^{n-1}q\left(q-a_{i}^{2}\right)$$

where k is a constant coefficient that determines the overall magnitude (or scaling) of the nonlinear force. In the context of potential energy, k controls the depth of the potential wells and the height of the potential barriers.

Alternatively, Equation (5) can be expanded and rearranged into a standard polynomial form in powers of q.

(6) $$F(q)=k_{1}q+k_{2}q^{3}+\cdots +k_{n}q^{2n-1}$$

Here, the coefficients $k_{1},k_{2},\cdots k_{n}$ are related to the parameters k and $a_{i}$ in Equation (5) through the binomial expansion. The polynomial representation in Equation (6) is more convenient for subsequent dynamic analysis using the averaging method, as it allows direct application of standard harmonic balance techniques.

From Equations (2), (3) and (6), the following can be deduced:

(7) $$S(q)=\sqrt{\frac{k_{1}-K_{1}}{K_{h}}q^{2}+\frac{k_{2}-K_{2}}{2K_{h}}q^{4}+\cdots +\frac{k_{n}}{nK_{h}}q^{2n}+x_{0}^{2}}-x_{0}$$

Equations (4) and (7) establish the functional relationship between the number of stable equilibrium points of the cantilever beam and the cam contour curve. Therefore, provided that the stiffness of the cantilever beam is known, the desired multistable vibration energy harvester can be realized by appropriately designing the cam contour curve.

## 3. Design of Tristable and Pentastable Energy Harvesters

This section presents the design of tristable and pentastable energy harvesters. The harvester parameters are listed in Table 1. The piezoelectric beam’s equivalent bending stiffness K is approximately 7.75 Nm^−1^. The measured stiffness $K_{h}$ is about 82.1 Nm^−1^, with pre-compression $x_{0}$ = 10 mm. The bearing radius is 2 mm.

A TEH and a PEH with specified equilibrium point coordinates are designed. The parameters in Table 1 were selected according to the available piezoelectric cantilever dimensions, material properties, the measured beam stiffness, and the feasible pre-compression range of the spring mechanism. The equilibrium point coordinates in Table 2 were not obtained through global optimization; instead, they were prescribed to demonstrate the design method and to enable a clear comparison between tristable and pentastable configurations. For this comparison, the maximum stable equilibrium coordinate of the PEH is set equal to that of the TEH, while the additional PEH equilibrium points are interpolated between the origin and the maximum coordinate.

**Figure 3:**
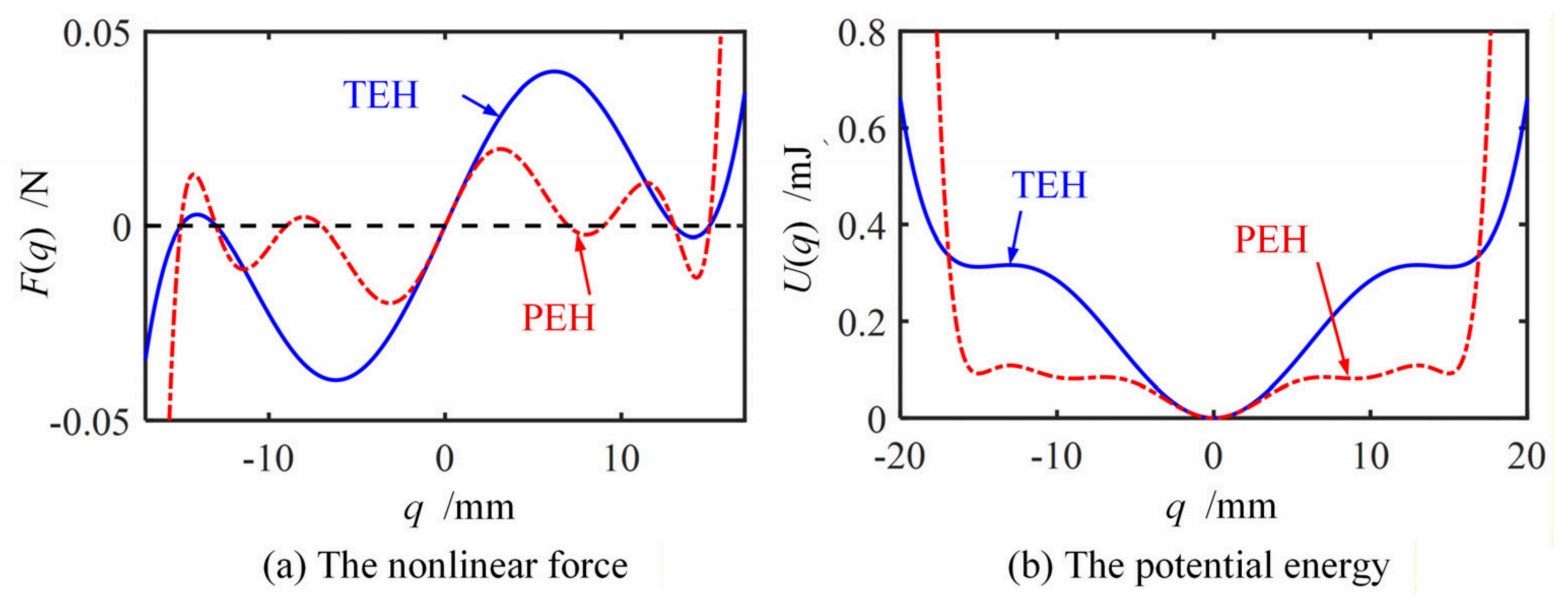
Comparison of nonlinear force and potential energy for the TEH and PEH.

Figure 3 compares the nonlinear forces and potential energies of the TEH and PEH. As the number of equilibrium points increases, the potential difference decreases, facilitating large inter-well motion. For example, the maximum potential difference is 0.32 mJ for the TEH and 0.11 mJ for the PEH.

**Figure 4:**
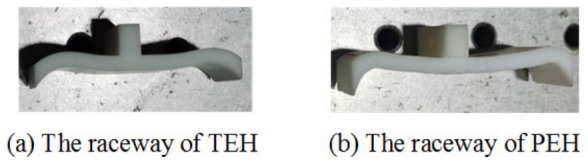
Two different processing raceways.

**Figure 5:**
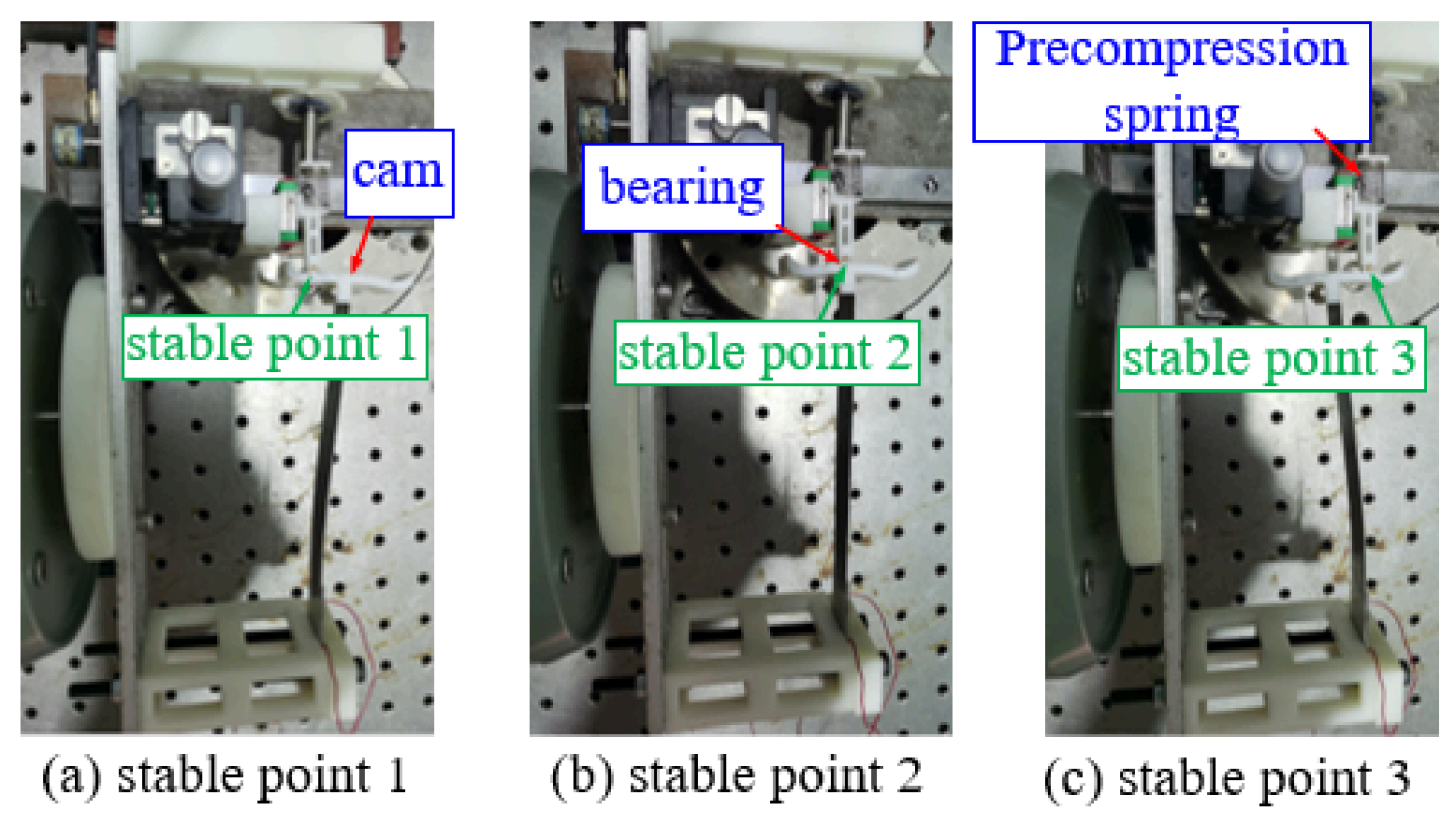
Three stable equilibrium points of the TEH.

**Figure 6:**
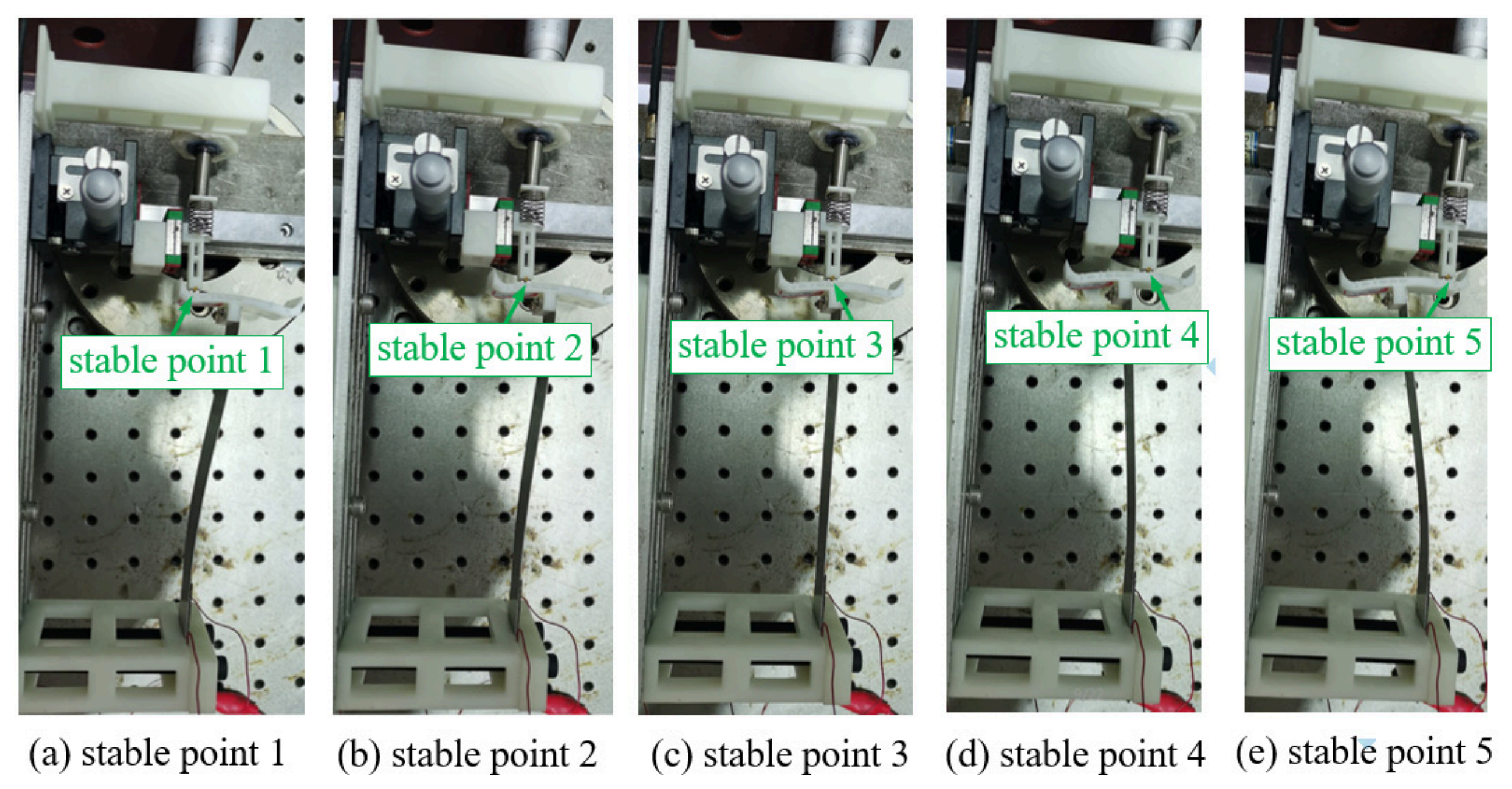
Five stable equilibrium points of the PEH.

Using Equations (4) and (7), the TEH and PEH raceways are 3D printed (Form3, Formlabs, Shanghai, China, Figure 4). By design, the two raceways have the same mass of about 5.14 g. As illustrated in Figure 5 and Figure 6, the TEH possesses three stable equilibrium points, and the PEH possesses five, which verifies the feasibility of the design.

## 4. Simulation and Experiment

### 4.1. The Average Method

After considering the nonlinear forces, Equation (1) can be rewritten as follows:

(8) $$\begin{array}{c} M\ddot{q}+C\dot{q}+k_{1}q+k_{2}q^{3}+\cdots +k_{n}q^{2n-1}-\theta V=mA_{b}\omega^{2}\cos(\omega t)\\ \theta \dot{q}+C_{p}\dot{V}+VR_{l}^{-1}=0 \end{array}$$

To generalize the analysis, we introduce the following dimensionless variables and parameters, following the approach in Ref. [62]:

(9) $$\begin{array}{l} q_{0}=\sqrt{\frac{\left|k_{1}\right|}{\left|k_{2}\right|}},\;\bar{q}=\frac{q}{q_{0}},\;\bar{t}=\omega_{n}t,\;\omega_{n}=\sqrt{\frac{\left|k_{1}\right|}{M}},\;\bar{V}=-\frac{C_{p}}{\theta q_{0}}V,\;\xi =\frac{C}{2M\omega_{n}}\\ \bar{\omega }=\frac{\omega }{\omega_{n}},\;F=\frac{mA_{b}\omega^{2}}{Mq_{0}\omega_{n}^{2}},\;\alpha =C_{p}\omega_{n}R_{l},\;\Gamma =\frac{\theta^{2}}{\left|k_{1}\right|C_{p}},\;\gamma_{i}=\frac{{\left|k_{1}\right|}^{i-2}k_{i}}{{\left|k_{2}\right|}^{i-1}},\;i=1,2,\cdots ,n \end{array}$$

Here, $q_{0}$ is a reference displacement; $\omega_{n}$ is the natural frequency; ξ is the damping ratio; $\bar{\omega }$ is the dimensionless excitation frequency; F is the dimensionless excitation amplitude; α is the dimensionless time constant of the piezoelectric circuit; and Γ is the dimensionless electromechanical coupling coefficient.

Substituting the above dimensionless variables into Equation (8) yields the following:

(10) $$\begin{array}{c} \ddot{\bar{q}}+2\xi \dot{\bar{q}}+\sum_{i=1}^{n}\gamma_{i}{\bar{q}}^{2i-1}+\Gamma \bar{V}=F\cos(\bar{\omega }\bar{t})\\ \alpha \dot{\bar{V}}+\bar{V}=\alpha \dot{\bar{q}} \end{array}$$

where the dots now denote differentiation with respect to the dimensionless time $\bar{t}$.

For the steady-state response, we assume that the displacement and voltage can be expressed as the sum of a constant term and a first harmonic term:

(11) $$\bar{q}=A_{0}+A_{1}\cos(\psi_{1}),\;\dot{\bar{q}}=-A_{1}\bar{\omega }\sin(\psi_{1})$$

(12) $$\bar{V}=A_{2}\cos(\psi_{2}),\;\dot{\bar{V}}=-A_{2}\bar{\omega }\sin(\psi_{2})$$

where $\psi_{1}=\bar{\omega }\bar{t}+\theta_{1},\psi_{2}=\bar{\omega }\bar{t}+\theta_{2}$ are the phase angles; $A_{1}$ and $A_{2}$ are the displacement and voltage amplitudes; and $\theta_{1}$ and $\theta_{2}$ are the corresponding phase shifts.

Substituting Equations (11) and (12) into the second equation of Equation (10), one obtains the following:

(13) $$-\alpha \bar{\omega }A_{2}\sin(\psi_{2})+A_{2}\cos(\psi_{2})=-\alpha \bar{\omega }A_{1}\sin(\psi_{1})$$

Differentiating Equation (13) and neglecting all derivative terms under steady-state conditions, we obtain the following:

(14) $$-\alpha {\bar{\omega }}^{2}A_{2}\cos(\psi_{2})-\bar{\omega }A_{2}\sin(\psi_{2})=-\alpha {\bar{\omega }}^{2}A_{1}\cos(\psi_{1})$$

From Equations (13) and (14), one obtains the following:

(15) $$\bar{V}=A_{2}\cos(\psi_{2})=\frac{\alpha^{2}{\bar{\omega }}^{2}A_{1}\cos(\psi_{1})-\alpha \bar{\omega }A_{1}\sin(\psi_{1})}{1+\alpha^{2}{\bar{\omega }}^{2}}$$

Further, Equation (15) can be expressed as follows:

(16) $$\bar{V}=\frac{k_{eq}}{\Gamma }A_{1}\cos(\psi_{1})-\frac{c_{eq}}{\Gamma }\bar{\omega }A_{1}\sin(\psi_{1})$$

Here, $k_{eq}$ and $c_{eq}$ are equivalent stiffness and equivalent damping caused by electric coupling, which can be expressed as follows:

(17) $$k_{eq}=\frac{\Gamma \alpha^{2}{\bar{\omega }}^{2}}{1+\alpha^{2}{\bar{\omega }}^{2}},\;c_{eq}=\frac{\Gamma \alpha }{1+\alpha^{2}{\bar{\omega }}^{2}}$$

From Equation (15), it is known that, the amplitudes of the voltage can be expressed as follows:

(18) $$\bar{V}=\frac{\alpha \bar{\omega }A_{1}}{\sqrt{1+\alpha^{2}{\bar{\omega }}^{2}}}$$

Substituting Equation (11) into the nonlinear term of Equation (10), one obtains the following:

(19) $$\sum_{i=1}^{n}\gamma_{i}{\bar{q}}^{2i-1}=\sum_{i=1}^{n}\gamma_{i}{\left(A_{0}+A_{1}\cos(\psi_{1})\right)}^{2i-1}$$

According to the binomial theorem, one obtains the following:

(20) $$\sum_{i=1}^{n}\gamma_{i}{\bar{q}}^{2i-1}=\sum_{i=1}^{n}\gamma_{i}\sum_{m=0}^{2i-1}C_{2i-1}^{m}A_{0}^{2i-1-m}A_{1}^{m}\cos{\left(\psi_{1}\right)}^{m}$$

From the Euler formula, the following can be deduced:

(21) $$\cos{\left(\psi_{1}\right)}^{m}={\left(\frac{e^{i\psi_{1}}+e^{-i\psi_{1}}}{2}\right)}^{m}=\frac{1}{2^{m}}\sum_{j=0}^{m}C_{m}^{j}e^{i(m-2j)\psi_{1}}$$

(22) $$\cos{\left(-\psi_{1}\right)}^{m}={\left(\frac{e^{-i\psi_{1}}+e^{i\psi_{1}}}{2}\right)}^{m}=\frac{1}{2^{m}}\sum_{j=0}^{m}C_{m}^{j}e^{-i(m-2j)\psi_{1}}$$

Adding Equations (21) and (22), one obtains the following:

(23) $$\cos{\left(\psi_{1}\right)}^{m}=\frac{1}{2^{m}}\sum_{j=0}^{m}C_{m}^{j}\frac{e^{i(m-2j)\psi_{1}}+e^{-i(m-2j)\psi_{1}}}{2}=\frac{1}{2^{m}}\sum_{j=0}^{m}C_{m}^{j}\cos\left((m-2j)\psi_{1}\right)$$

As can be seen from Equation (23), when m is odd and $j=\left(m\pm 1\right)/2$, the first harmonic term can be obtained; when m is an even number and j=m/2, a constant term is obtained. Therefore, if only the first harmonic term and the constant term are considered, one obtains the following:

(24) $$\sum_{i=1}^{n}\gamma_{i}{\bar{q}}^{2i-1}\approx \sum_{i=1}^{n}\sum_{m=0}^{2i-1}\frac{\gamma_{i}C_{2i-1}^{m}A_{0}^{2i-1-m}A_{1}^{m}C_{m}^{\frac{m+1}{2}}}{2^{m-1}}\cos(\psi_{1})+\sum_{i=1}^{n}\sum_{m=0}^{2i-1}\frac{\gamma_{i}C_{2i-1}^{m}A_{0}^{2i-1-m}A_{1}^{m}C_{m}^{\frac{m}{2}}}{2^{m}}$$

It is assumed that m=2h−1 or m=2h−2. Therefore, Equation (24) can be rewritten as follows:

(25) $$\sum_{i=1}^{n}\gamma_{i}{\bar{q}}^{2i-1}=H_{1}\cos(\psi_{1})+H_{2}$$

where

(26) $$H_{1}=\sum_{i=1}^{n}\sum_{h=1}^{i}\frac{\gamma_{i}C_{2i-1}^{2h-1}C_{2h-1}^{h}A_{0}^{2i-2h}A_{1}^{2h-1}}{2^{2h-2}}$$

(27) $$H_{2}=\sum_{i=1}^{n}\sum_{h=1}^{i}\frac{\gamma_{i}C_{2i-1}^{2h-2}C_{2h-2}^{h-1}A_{0}^{2i-2h+1}A_{1}^{2h-2}}{2^{2h-2}}$$

Using the method of variation of constant [63], it is assumed that both $A_{1}$ and $\theta_{1}$ are functions of time $\bar{t}$. Differentiating Equation (11) with respect to $\bar{t}$, one obtains the following:

(28) $$\begin{array}{l} \dot{\bar{q}}=\left(-A_{1}\bar{\omega }-A_{1}{\dot{\theta }}_{1}\right)\sin(\psi_{1})+{\dot{A}}_{1}\cos(\psi_{1})\\ \ddot{\bar{q}}=-{\dot{A}}_{1}\bar{\omega }\sin(\psi_{1})-\left(A_{1}\bar{\omega }{\dot{\theta }}_{1}+{\bar{\omega }}^{2}A_{1}\right)\cos(\psi_{1}) \end{array}$$

Substituting Equations (16), (25) and (28) into the first equation of Equation (10), solving the algebra equation with respect to ${\dot{A}}_{1}$ and ${\dot{\theta }}_{1}$, one obtains the following:

(29) $$H_{2}=0$$

(30) $$\begin{array}{l} {\dot{A}}_{1}=-\frac{\sin(\psi_{1})\left(\left(({\bar{\omega }}^{2}-k_{eq})A_{1}-H_{1})\cos(\psi_{1})+(2\xi +c_{eq})\bar{\omega }A_{1}\sin(\psi_{1})+F\cos(\bar{\omega }\bar{t}\right)\right)}{\bar{\omega }}\\ {\dot{\theta }}_{1}=-\frac{\cos(\psi_{1})\left(\left(({\bar{\omega }}^{2}-k_{eq})A_{1}-H_{1})\cos(\psi_{1})+(2\xi +c_{eq})\bar{\omega }A_{1}\sin(\psi_{1})+F\cos(\bar{\omega }\bar{t}\right)\right)}{A_{1}\bar{\omega }} \end{array}$$

From the principle of averaging method, it is assumed that $A_{1}$ and $\theta_{1}$ vary much more slowly with $\bar{t}$ than $\psi_{1}$. This enables us to average out the variations of $\psi_{1}$ in Equation (30). The averaging equation of amplitude $A_{1}$ and $\theta_{1}$ is rewritten as follows:

(31) $$\begin{array}{l} {\dot{A}}_{1}=-\frac{\int_{0}^{2\pi }\left(\left(({\bar{\omega }}^{2}-k_{eq})A_{1}-H_{1})\cos(\psi_{1})+(2\xi +c_{eq})\bar{\omega }A_{1}\sin(\psi_{1})+F\cos(\bar{\omega }\bar{t}\right)\right)\sin(\psi_{1})d\psi_{1}}{2\pi \bar{\omega }}\\ {\dot{\theta }}_{1}=-\frac{\int_{0}^{2\pi }\left(\left(({\bar{\omega }}^{2}-k_{eq})A_{1}-H_{1})\cos(\psi_{1})+(2\xi +c_{eq})\bar{\omega }A_{1}\sin(\psi_{1})+F\cos(\bar{\omega }\bar{t}\right)\right)\cos(\psi_{1})d\psi_{1}}{2\pi A_{1}\bar{\omega }} \end{array}$$

From Equation (31), one obtains the following:

(32) $$\begin{array}{c} \frac{dA_{1}}{dt}=-\frac{F\sin(\theta_{1})+(2\xi +c_{eq})\bar{\omega }A_{1}}{2\bar{\omega }}\\ A_{1}\frac{d\theta_{1}}{dt}=-\frac{F\cos(\theta_{1})+({\bar{\omega }}^{2}-k_{eq})A_{1}-H_{1}}{2\bar{\omega }} \end{array}$$

Steady periodic motions occur when ${\dot{A}}_{1}=0$ and ${\dot{\theta }}_{1}=0$. Hence, the amplitude–frequency response relationship is derived using the following formula:

(33) $${\left(k_{eq}+\frac{H_{1}}{A_{1}}-{\bar{\omega }}^{2}\right)}^{2}+{\left(2\xi \bar{\omega }+c_{eq}\bar{\omega }\right)}^{2}=\frac{F^{2}}{A_{1}^{2}}$$

The stability of the steady-state motion is determined by investigating the nature of the singular points of Equation (32). To accomplish this, we note the following:

(34) $$A_{1}=A_{1}^{*}+\Delta A_{1},\;\theta_{1}=\theta_{1}^{*}+\Delta \theta_{1}$$

Substituting Equation (34) into Equation (32), expanding for small $\Delta A_{1}$ and $\Delta \theta_{1}$, noting that $A_{1}^{*}$ and $\theta_{1}^{*}$ satisfy Equation (33), and keeping linear terms in $\Delta A_{1}$ and $\Delta \theta_{1}$, one obtains the following:

(35) $$\begin{array}{l} \frac{d\Delta A_{1}}{dt}=-\frac{2\xi +c_{eq}}{2}\Delta A_{1}+\frac{A_{1}\left(\omega^{2}-k_{eq}\right)-H_{1}}{2\omega }\Delta \theta_{1}\\ \frac{d\Delta \theta_{1}}{dt}=-\frac{\omega^{2}-k_{eq}-\frac{\partial H_{1}}{\partial A_{1}}}{2A_{1}\omega }\Delta A_{1}-\frac{2\xi +c_{eq}}{2}\Delta \theta_{1} \end{array}$$

Thus, the stability of the steady-state motions depends on the eigenvalues of the coefficient matrix on the right-hand sides of Equation (35). The following eigenvalue equation can be obtained:

(36) $$\left|\begin{array}{cc} -\frac{2\xi +c_{eq}}{2}-\lambda & \frac{A_{1}\left(\omega^{2}-k_{eq}\right)-H_{1}}{2\omega }\\ -\frac{\omega^{2}-k_{eq}-\frac{\partial H_{1}}{\partial A_{1}}}{2A_{1}\omega } & -\frac{2\xi +c_{eq}}{2}-\lambda \end{array}\right|=0$$

Expanding this determinant, one obtains the following:

(37) $$\lambda^{2}+\left(2\xi +c_{eq}\right)\lambda +{\left(\frac{2\xi +c_{eq}}{2}\right)}^{2}+\frac{\left(A_{1}\omega^{2}-A_{1}k_{eq}-H_{1}\right)\left(\omega^{2}-k_{eq}-\frac{\partial H_{1}}{\partial A_{1}}\right)}{4A_{1}\omega^{2}}=0$$

Hence, the steady-state motions are unstable when the following occurs.

(38) $${\left(\frac{2\xi +c_{eq}}{2}\right)}^{2}+\frac{\left(A_{1}\omega^{2}-A_{1}k_{eq}-H_{1}\right)\left(\omega^{2}-k_{eq}-\frac{\partial H_{1}}{\partial A_{1}}\right)}{4A_{1}\omega^{2}}<0$$

Otherwise, they are stable.

### 4.2. Experiment

**Figure 7:**
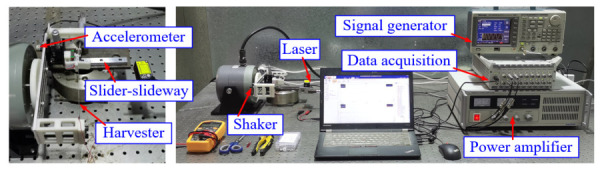
Experimental setup used for validation of the analytical model.

This section presents the experimental investigation; the experimental setup is illustrated in Figure 7. To eliminate gravitational effects, the energy harvester is mounted horizontally on a low-friction slider slideway (Mgn15c, HIWIN, Shanghai, China) and connected to a vibration exciter (JZK-10, SINOCERA PIEZOTRONICS, Shanghai, China). An acceleration sensor (B&K 4514, Shanghai, China) is attached to the harvester to monitor the base acceleration. A single-frequency sinusoidal signal generated by a signal generator (AFG3022C, Tektronix, Shanghai, China) is amplified using a power amplifier (YE5872A, SINOCERA PIEZOTRONICS, Shanghai, China) and then fed to the exciter. A laser displacement sensor (HG-C1100, Panasonic, Shanghai, China) is placed near the tip of the cantilever beam to measure its displacement response. The mass of the miniature bearing and its accessories is approximately 1.21 g.

A resistive load of 1 MΩ is used in the main frequency–response tests. Single-frequency excitation is applied throughout the experiment. The measured response at each frequency is subjected to fast Fourier transform (FFT), and the amplitude at the excitation frequency is extracted and compared with simulation results. The time-domain tip displacement signals under representative harmonic excitations are also retained to verify whether the harvester undergoes intra-well or inter-well motion. Two methods are employed in simulations. The first solves the governing Equation (10) using the Runge–Kutta method with up-sweep and down-sweep analyses; the second obtains an approximate analytical solution using the averaging method (as given in Equation (33)).

**Figure 8:**
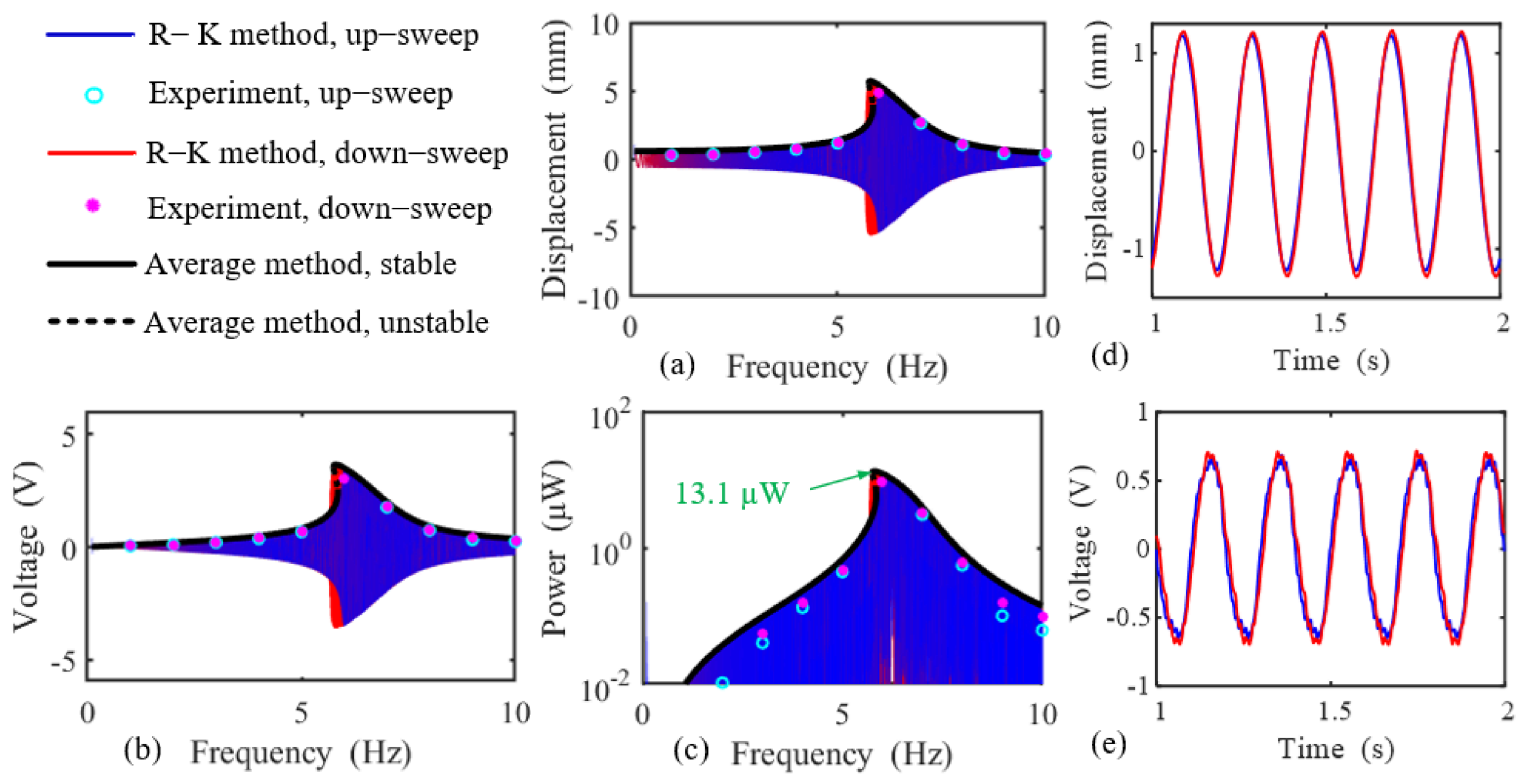
Comparison of simulation and experiment of the TEH at 0.1 g: (**a**) displacement response; (**b**) output voltage; (**c**) output power; (**d**) time-domain displacement at 5 Hz; (**e**) time-domain voltage at 5 Hz.

**Figure 9:**
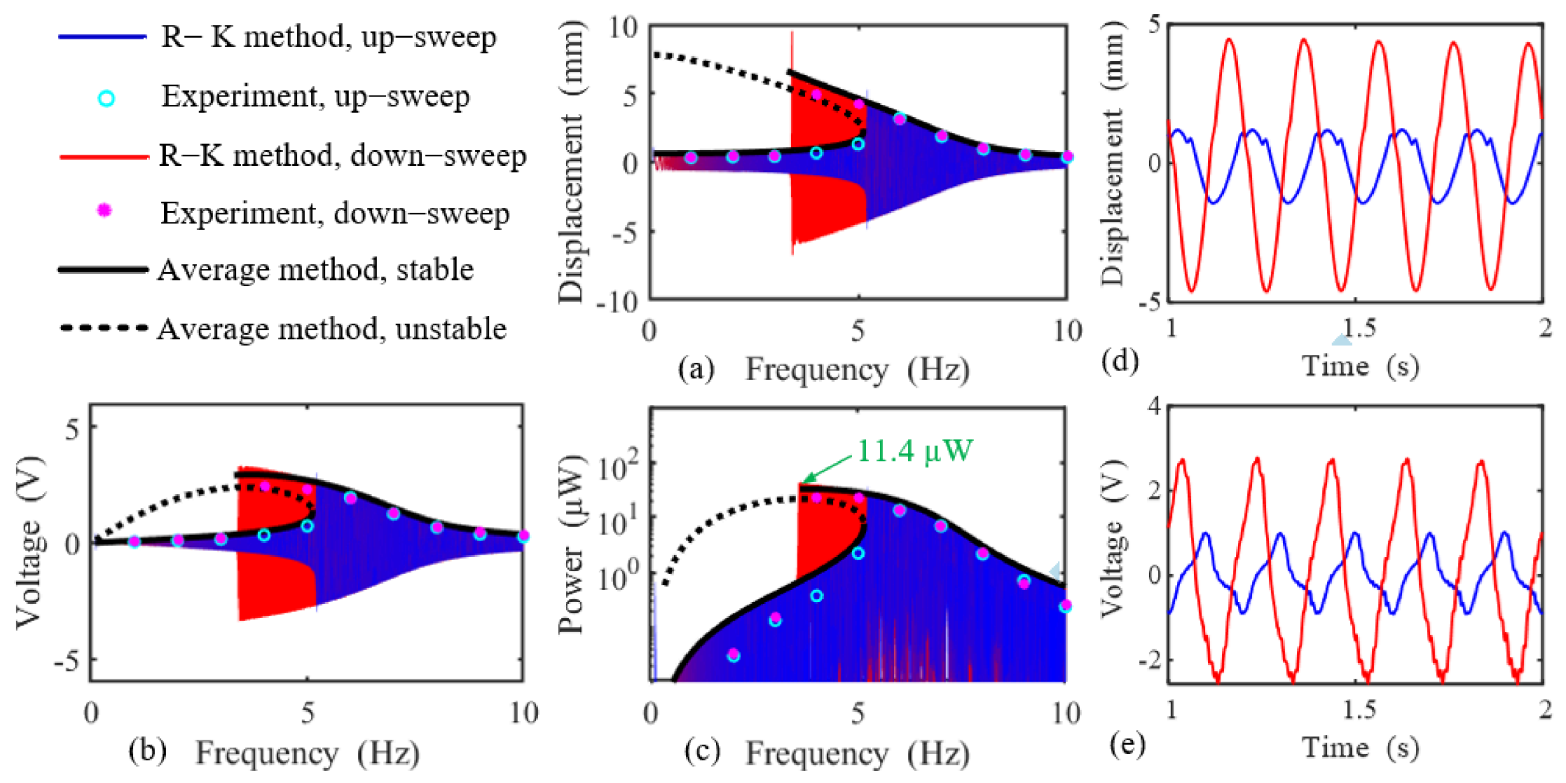
Comparison of simulation and experiment of the PEH at 0.1 g: (**a**) displacement response; (**b**) output voltage; (**c**) output power; (**d**) time-domain displacement at 5 Hz; (**e**) time-domain voltage at 5 Hz.

Figure 8 and Figure 9 show the displacement response, output voltage, and power of the two energy harvesters under an acceleration of 0.1 g (g = 9.8 m/s^2^). The results obtained using the Runge–Kutta method are in good agreement with those from the averaging method, confirming the correctness of the derived formulas. Moreover, the experimental results closely match the simulations, demonstrating the feasibility of the proposed energy harvester. The following conclusions can be drawn: (1) Compared with the TEH, the PEH exhibits a wider bandwidth and a lower harvesting frequency, which is beneficial for capturing low-frequency vibration energy. (2) Under very small excitation acceleration, neither the TEH nor the PEH can achieve large inter-well motion, and both display softening nonlinearity. In Equation (8), since $k_{1}$ > 0 and $k_{2}$ < 0 and the higher-order terms are negligible for small displacements, the system behaves as a softening nonlinear system.

**Figure 10:**
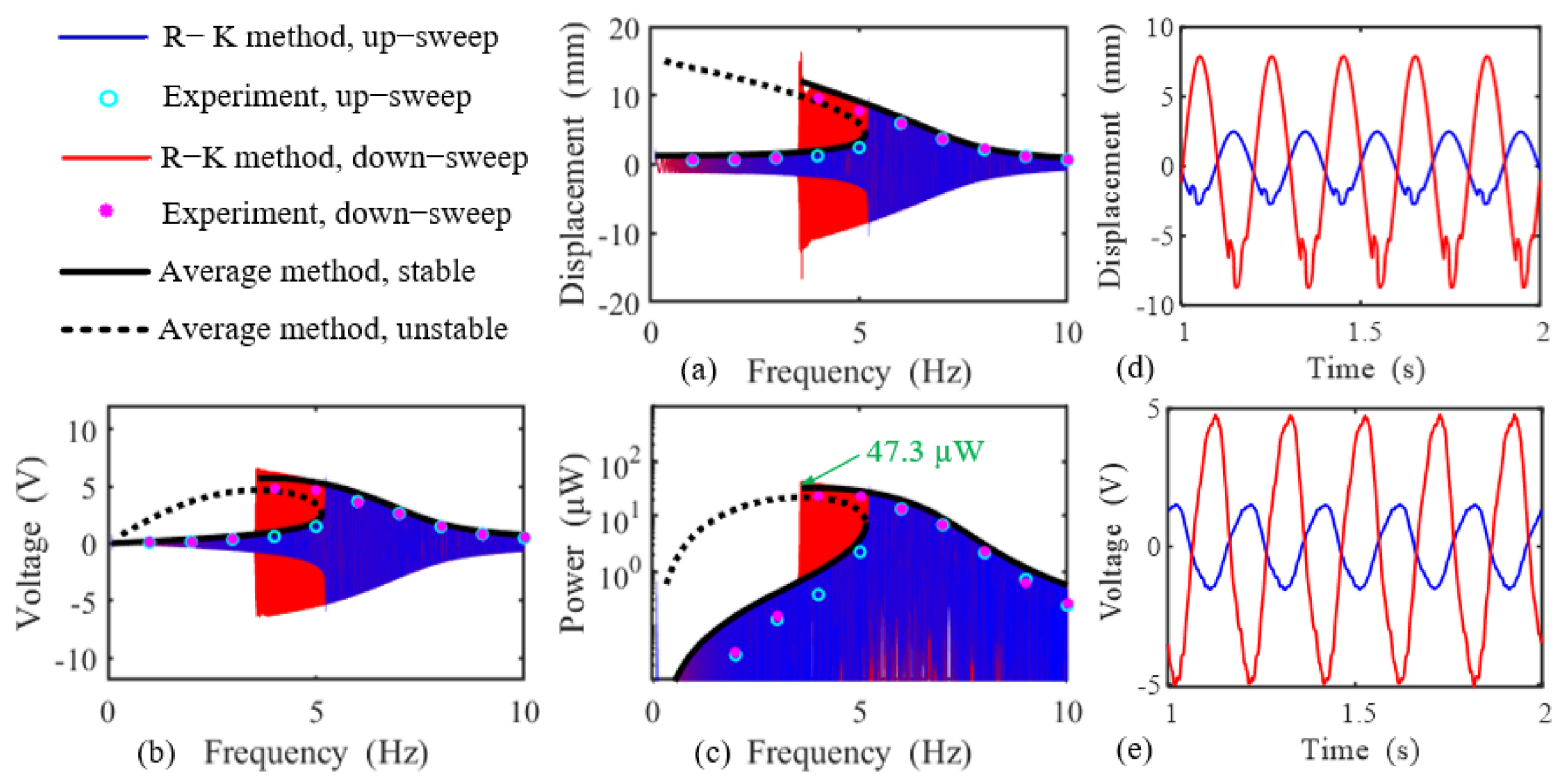
Comparison of simulation and experiment of the TEH at 0.2 g: (**a**) displacement response; (**b**) output voltage; (**c**) output power; (**d**) time-domain displacement at 5 Hz; (**e**) time-domain voltage at 5 Hz.

**Figure 11:**
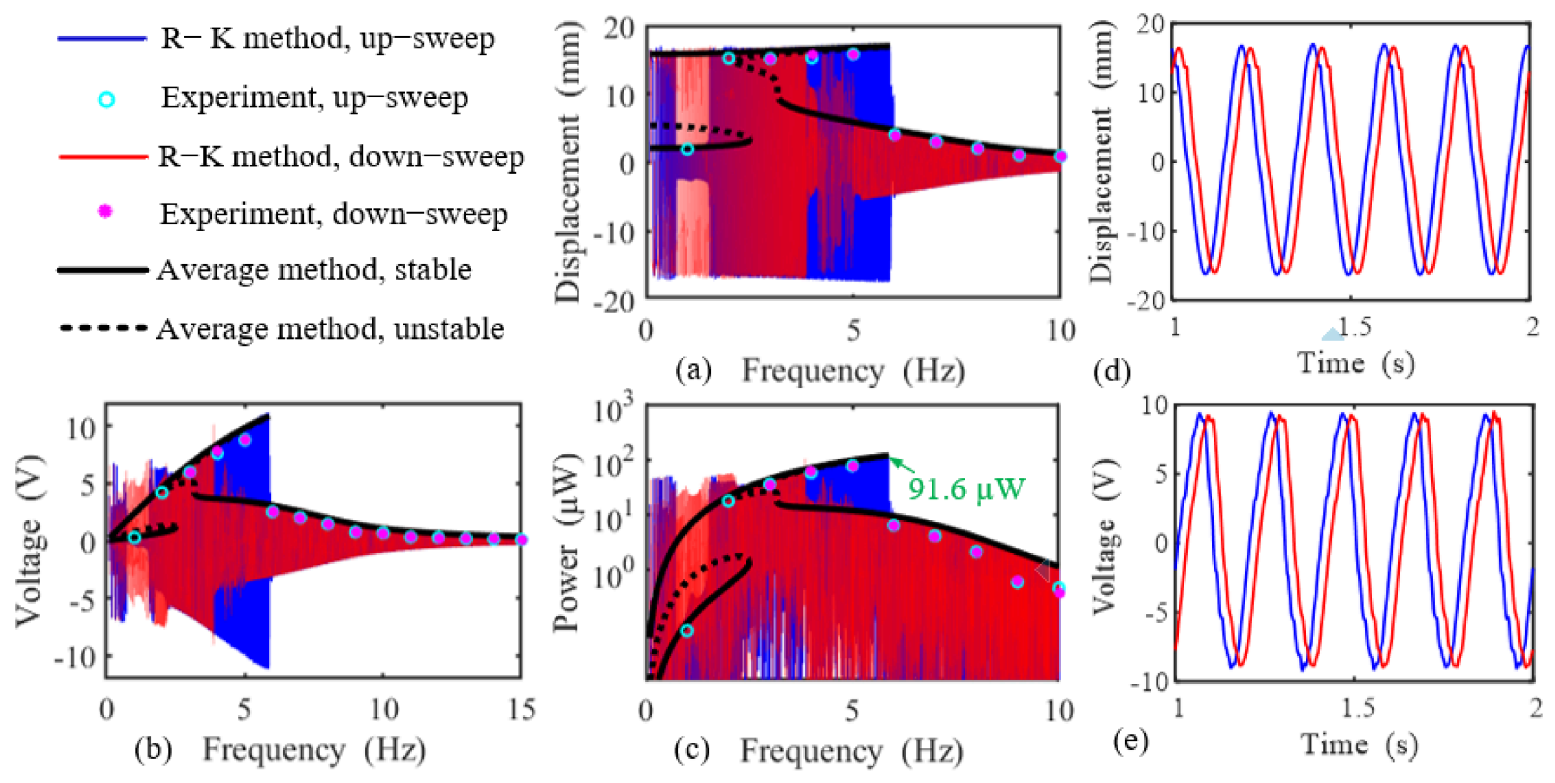
Comparison of simulation and experiment of the PEH at 0.2 g: (**a**) displacement response; (**b**) output voltage; (**c**) output power; (**d**) time-domain displacement at 5 Hz; (**e**) time-domain voltage at 5 Hz.

Figure 10 and Figure 11 show the displacement, output voltage, and power at 0.2 g. The TEH remains in intra-well motion, whereas the PEH can overcome the maximum potential barrier to perform inter-well vibrations. Accordingly, the PEH exhibits wider bandwidth, higher peak power, and hardening nonlinearity because the positive coefficient $k_{3}$ in Equation (8) becomes dominant as displacement increases.

It should be noted that for the PEH in the 0~2.5 Hz range, the averaging method produces larger errors than the Runge–Kutta method. This is because the averaging method assumes a single-harmonic response and provides only approximate solutions. In this frequency band, the system exhibits significant higher-order harmonics, which reduce the accuracy of the averaging approximation.

**Figure 12:**
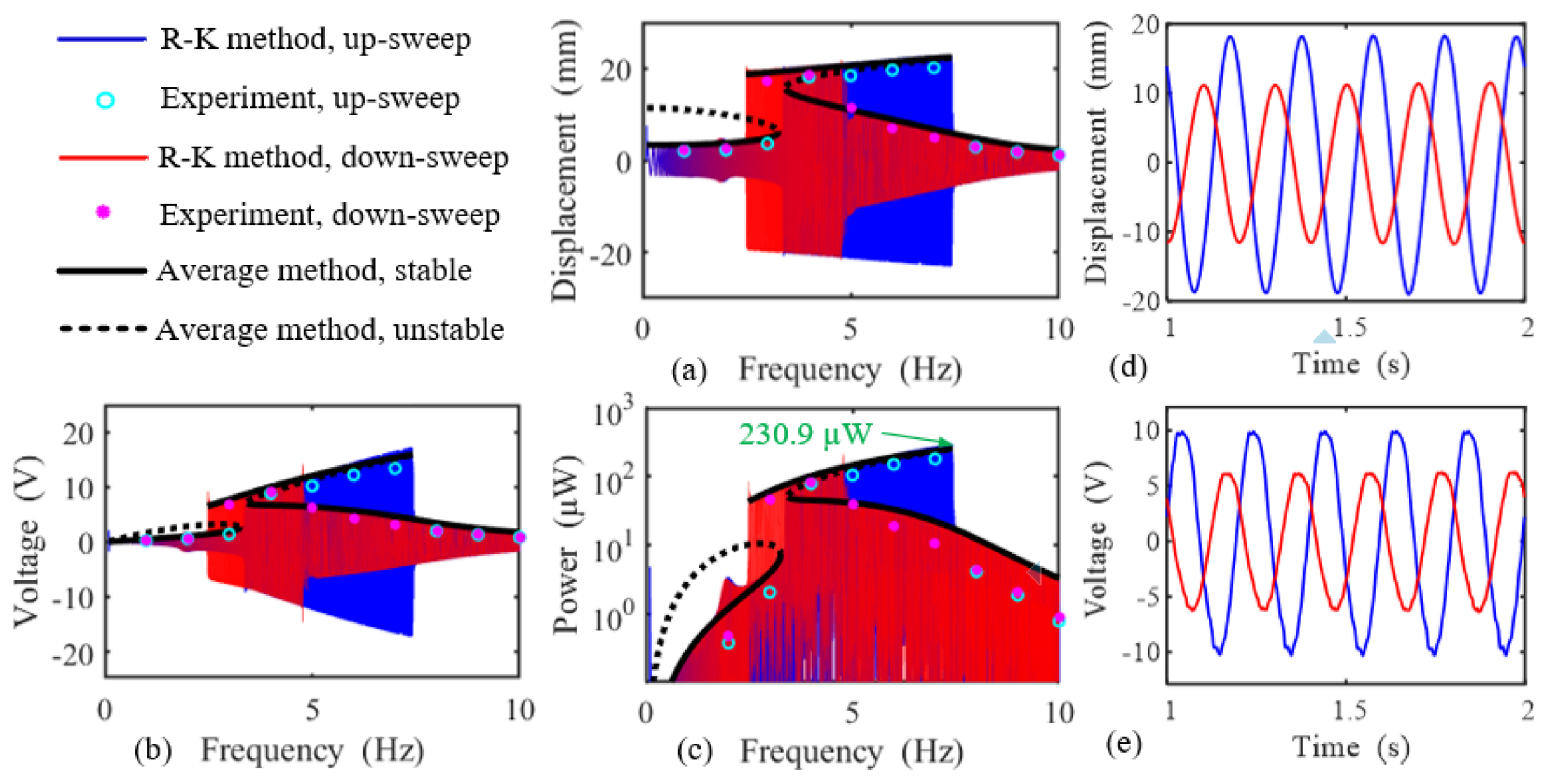
Comparison of simulation and experiment of the TEH at 0.4 g: (**a**) displacement response; (**b**) output voltage; (**c**) output power; (**d**) time-domain displacement at 5 Hz; (**e**) time-domain voltage at 5 Hz.

**Figure 13:**
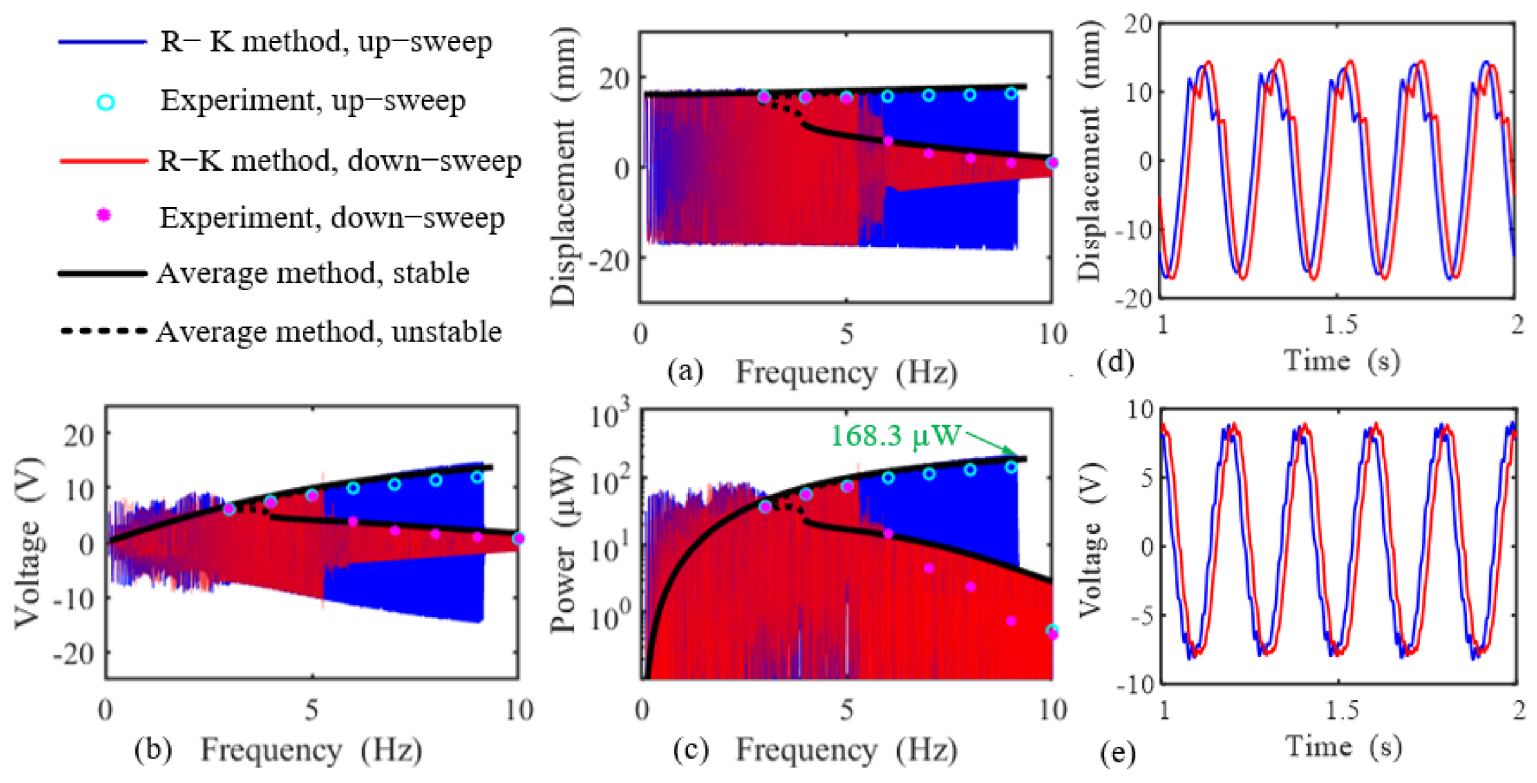
Comparison of simulation and experiment of the PEH at 0.4 g: (**a**) displacement response; (**b**) output voltage; (**c**) output power; (**d**) time-domain displacement at 5 Hz; (**e**) time-domain voltage at 5 Hz.

Figure 12 and Figure 13 present the displacement responses, output voltage, and power at 0.4 g. The TEH now also overcomes the potential barrier and undergoes large inter-well vibrations with hardening nonlinearity. Compared with the TEH, the PEH offers a wider bandwidth and a lower harvesting frequency, but lower peak power. This low-frequency operating capability is particularly meaningful for practical marine and mechanical systems. For instance, propulsion shafting can generate shaft frequency longitudinal vibration, which is typically below 3 Hz, and wave-induced excitation is often around 1 Hz. Therefore, the customized PEH may provide a potential energy-harvesting solution for low-duty cycle self-powered sensing in such low-frequency vibration environments [64,65].

Simulations and experiments thus confirm the feasibility of energy harvesters with user-specified equilibrium points. Existing multistable harvesters (with three or more stable points) are typically realized using multiple springs or magnets, e.g., a tristable harvester with five springs [29] or three magnets [66], a quadstable and a pentastable harvester with four [34] and five [67] magnets, respectively. As the number of equilibrium points increases, so does the number of required springs/magnets and the precision of their placement, leading to greater structural complexity. In contrast, the present method achieves multistability by designing different raceways; complexity does not scale with the number of equilibrium points. This is the first advantage. Furthermore, arbitrarily specifying the coordinates of equilibrium points is difficult with springs/magnets but straightforward in the proposed design stage, greatly facilitating further optimization. This is the second advantage.

Additionally, the performance of the designed energy harvesters (EHs) is compared with other reported EHs in the literature. The normalized power density (NPD) is employed to evaluate the output performance. As shown in Table 3, both the TEH and the PEH we designed require the lowest excitation level among the compared devices, which is beneficial for harvesting vibration energy under low-excitation conditions. Moreover, our harvesters exhibit favorable bandwidth and NPD. However, it should also be noted that the equilibrium point coordinates of the designed TEH and PEH were prescribed for demonstration rather than optimized; therefore, their performance may not yet be optimal. Consequently, further improvements are still possible through optimization of the equilibrium point coordinate distribution.

To clarify the relevance to self-powered sensor nodes, the harvested power was evaluated as the average output power delivered to the load resistor. The obtained power level was compared with representative low-power wireless sensing devices reported in the literature. This comparison is intended only as an indicative power-level assessment and suggests that the proposed harvester may serve as a vibration energy source for low-duty cycle microsensor applications when combined with suitable rectification, power-management, and energy storage circuits.

It should be emphasized that the present experimental validation focuses on the nonlinear dynamic response and energy-harvesting capability of the harvester, rather than on a complete self-powered sensor node demonstration. Rectification circuits, power management units, energy storage elements, and actual sensor node operation were not experimentally implemented in this study. In addition, repeatability, measurement uncertainty, friction and wear of the roller–raceway contact, fabrication tolerance of the 3D-printed raceway, and long-term durability may affect practical performance and require systematic investigation in future work.

## 5. Conclusions

In this study, a piezoelectric cantilever beam-based multistable vibration energy harvester with user-specified equilibrium points (both number and coordinates) is proposed. As examples, a tristable and a pentastable harvester are designed. Simulations and experiments confirm the feasibility and effectiveness of the proposed approach. The main conclusions are as follows:

(1) Under weak excitation (e.g., 0.1 g), the multistable system is dominated by softening nonlinearity and can only undergo intra-well motion.
(2) As the excitation increases (e.g., 0.4 g), the system can overcome the potential barrier to perform inter-well jumping and exhibits hardening nonlinearity.
(3) When the acceleration is sufficiently large for both the tristable and pentastable harvesters to undergo inter-well vibration, the pentastable harvester exhibits a wider bandwidth but lower peak power, whereas the tristable harvester shows a narrower bandwidth but higher peak power.
(4) Moreover, appropriately increasing the number of equilibrium points lowers the critical excitation amplitude required for inter-well jumping, providing a general design guideline for broadband energy harvesting in low-excitation environments.
(5) The present results indicate potential for low-duty cycle microsensor power supply; however, practical sensor node operation will require further integration and experimental validation of rectification, power management, and energy-storage circuits.

## Figures and Tables

**Table 1:** Geometric and material parameters; the subscripts p and s represent the piezoelectric layer and the substructure layer, respectively.

Parameter	Piezoelectric Layer	Substrate Layer
Length, $L_{p}$, $L_{s}$ (mm)	10	135
Width, $b_{p}$, $b_{s}$ (mm)	19.8	19.8
Thickness, $h_{p}$, $h_{s}$ (mm)	0.21	0.36
Young’s modulus, $Y_{p}$, $Y_{s}$ (GPa)	56	69
Density, $\rho_{p}$, $\rho_{s}$ (kg/m^3^)	7500	2700
Piezoelectric constant, $d_{31}$ (pm/V)	−186	-
Permittivity, $\varepsilon_{33}^{\bar{S}}$ (F/m)	3400 $\varepsilon_{0}$	-

**Table 2:** The designed parameters of the TEH and the PEH.

Type	Linear Stiffness *k*_1_	Coordinates (mm)	Stable Equilibrium Points
TEH	10 Nm^−1^	0, $a_{1}=13$, $a_{2}=15$	0, ±15 mm
PEH	10 Nm^−1^	0, $a_{1}=7$, $a_{2}=9$, $a_{3}=13$, $a_{4}=15$	0, ±9 mm, ±15 mm

**Table 3:** Performance comparisons of recently reported energy harvesters.

Refs.	Type	Acc. (m/s^2^)	Operating Frequency Range (≥5 μW) (Hz)		NPD (μW/cm^3^/g^2^)
			Range	Bandwidth	
Li et al. [68]	Monostable	10	22~26	4	5.5
Zhou et al. [69]	Bistable	3.5	8~14	6	26.1
Lallart et al. [70]	Tristable	5	8~12	4	4.77
Lai et al. [71]	Tristable	3	2~10	8	225.7
Kim and Seok [72]	Quadstable	15	15~35	20	4.96
This work, TEH	Tristable	2	3.5~7.6	4.1	17.4
		4	2.5~9.3	6.8	34.9
This work, PEH	Pentastable	2	0.5~7.6	7.1	28.2
		4	0.9~9.1	8.2	21.3

## Data Availability

The original contributions presented in this study are included in the article. Further inquiries can be directed to the corresponding author.
